# Does directly observed therapy improve tuberculosis treatment? More evidence is needed to guide tuberculosis policy

**DOI:** 10.1186/s12879-016-1862-y

**Published:** 2016-10-04

**Authors:** Zoë M. McLaren, Amanda A. Milliken, Amanda J. Meyer, Alana R. Sharp

**Affiliations:** School of Public Health, University of Michigan, Ann Arbor, USA

**Keywords:** Tuberculosis, Directly observed therapy short course (DOTS), Evidence-based medicine, Patient-centered care

## Abstract

**Background:**

Tuberculosis (TB) now ranks alongside HIV as the leading infectious disease cause of death worldwide and incurs a global economic burden of over $12 billion annually. Directly observed therapy (DOT) recommends that TB patients complete the course of treatment under direct observation of a treatment supporter who is trained and overseen by health services to ensure that patients take their drugs as scheduled. Though the current WHO End TB Strategy does not mention DOT, only “supportive treatment supervision by treatment partners”, many TB programs still use it despite the fact that the has not been demonstrated to be statistically significantly superior to self-administered treatment in ensuring treatment success or cure.

**Discussion:**

DOT is designed to promote proper adherence to the full course of drug therapy in order to improve patient outcomes and prevent the development of drug resistance. Yet over 8 billion dollars is spent on TB treatment each year and thousands undergo DOT for all or part of their course of treatment, despite the absence of rigorous evidence supporting the superior effectiveness of DOT over self-administration for achieving drug susceptible TB (DS-TB) cure. Moreover, the DOT component burdens patients with financial and opportunity costs, and the potential for intensified stigma.

To rigorously evaluate the effectiveness of DOT and identify the essential contributors to both successful treatment and minimized patient burden, we call for a pragmatic experimental trial conducted in real-world program settings, the gold standard for evidence-based health policy decisions. It is time to invest in the rigorous evaluation of DOT and reevaluate the DOT requirement for TB treatment worldwide.

**Summary:**

Rigorously evaluating the choice of treatment supporter, the frequency of health care worker contact and the development of new educational materials in a real-world setting would build the evidence base to inform the optimal design of TB treatment protocol. Implementing a more patient-centered approach may be a wise reallocation of resources to raise TB cure rates, prevent relapse, and minimize the emergence of drug resistance. Maintaining the status quo in the absence of rigorous supportive evidence may diminish the effectiveness of TB control policies in the long run.

## Background

Tuberculosis (TB) now ranks alongside HIV as the leading infectious disease cause of death worldwide and incurs a global economic burden of more than $12 billion annually [1]. In 2014, approximately 9.6 million people contracted TB globally and 1.5 million died from the disease [1]. Directly observed therapy (DOT), based on standardized TB treatment with supervision and patient support, was pioneered in the 1970s and was a core component of the World Health Organization (WHO) TB strategy from 1996 to 2015 [2]. Though the current WHO End TB Strategy does not mention DOT but rather “supportive treatment supervision by treatment partners”, many TB programs still use DOT despite the fact that the has not been demonstrated to be statistically significantly superior to self-administered treatment in ensuring treatment success or cure [3].

The DOT component, which accounts for up to 75 % of the provider costs of TB treatment [4], recommends that patients complete the course of treatment under direct observation of a treatment partner or supporter who is trained and overseen by health services to ensure that patients take their drugs as scheduled [2]. DOT is designed to promote proper adherence to the full course of drug therapy in order to improve patient outcomes and prevent the development of drug resistance. Yet over 8 billion dollars is spent on TB treatment each year [1] and thousands undergo DOT for all or part of their course of treatment, despite the absence of rigorous evidence supporting the superior effectiveness of DOT over self-administration for achieving drug susceptible TB (DS-TB) cure [5–8]. Moreover, the DOT component burdens patients with financial and opportunity costs, and the potential for intensified stigma [4, 9, 10].

Thus, the effectiveness of DOT for DS-TB should be rigorously evaluated relative to less burdensome alternative treatment protocols with a pragmatic experimental trial conducted in real-world program settings, the gold standard foundation for evidence-based health policy decisions. Self-administration could prove to be not only as *effective* as DOT in curing TB, preventing relapse and minimizing the emergence of drug resistance, but also more *cost-effective*, because it is generally less expensive than health-center-based DOT [4]. Self-administration could therefore relieve the burden on TB patients while increasing cost-effectiveness and protecting public health. Considering the overall financial and human cost of TB and the clinical equipoise of DOT, the relative cost of an experimental evaluation of the effectiveness of DOT would be a wise investment of limited funds.

Between 1995 and 2012, an estimated 56 million individuals were successfully treated for tuberculosis under the DOTS/Stop TB strategy in 184 countries [11]. Worldwide, approximately 86 % of TB patients successfully complete treatment; however, treatment success and cure rates vary widely by geographic region and per capita income and TB cure rates may be substantially lower than treatment success rates [1]. Considering the worldwide 17 % case fatality rate and cure rates as low as 69 % in Russia and 72 % in Brazil [1], it is imperative that TB treatment protocols be subject to rigorous evaluation of their effectiveness in curing DS-TB, preventing relapse and minimizing the emergence of drug resistance.

## The burden of direct observation

The financial and psychosocial burden of DOT on patients can be substantial, even when national TB programs provide drugs at no direct cost to the patient [4]. In DOT programs requiring patients to make multiple clinic (or community center) visits per week, patients may incur significant monetary and time costs of travel if reimbursements or subsidies to offset these costs are not provided [12]. Over the course of 6 or more months of TB treatment these costs add up. Although some TB treatment programs have health workers conduct DOT in patients’ homes rather than health facilities, no official numbers exist on how many programs administer TB treatment through this method [1]. Aside from direct costs, frequent visits to health facilities for DOT may interfere with a patient’s work schedule or home production responsibilities (such as child care), lead to lost wages, or cause the loss of employment [12]. In addition, frequent visits to TB treatment facilities or from DOT health workers may intensify the stigma associated with TB and diminish a patient’s ability to maintain privacy about their health [9]. Fear of stigma and the burden of DOT may prevent patients from completing TB treatment, or seeking TB testing in the first place [9].

## Limited high-quality evidence of DOT effectiveness

In reviewing the recent literature on DOT in developing countries we find that there is a very limited high-quality evidence demonstrating that DOT is more effective than self-administration at achieving DS-TB cure. We identified four systematic reviews [5–8] that compared DOT to self-administration. Among these reviews, Tian et al. [6] found no convincing evidence that DOT achieves statistically significantly higher treatment success or cure rates than self-administration when the two regimens were compared in four randomized controlled trials (RCTs). Data from eight observational studies suggested DOT to be more effective, however, these non-randomized studies do not adequately address concerns about confoundedness [6]. Pasipanodya & Gumbo’s [5] meta-analysis of five RCTs and five observational studies demonstrated that DOT was no more effective at preventing treatment failure than self-administration. One systematic review [8] concluded that DOT improved treatment success and cure rates based on results from one RCT and two quasi-experimental studies. Of the six RCTs examined by Karumbi & Garner [7], only one additional study found higher cure and treatment completion rates among patients enrolled in DOT when compared to self-administration. Based on the preponderance of high-quality RCT evidence, we echo the conclusions from systematic reviews finding that the evidence provides “no assurance that DOT compared with self-administration of treatment has any quantitatively important effect on cure or treatment completion” [13].

## A call for rigorous evaluation of DOT

To rigorously evaluate the effectiveness of DOT and identify the essential contributors to both successful treatment and minimized patient burden, we call for a pragmatic experimental trial conducted in real-world program settings. Experimental trials have frequently been used to guide and evaluate public health interventions such as the effect of immediate initiation of ART treatment for HIV patients [14], a pre-eclampsia intervention on maternal mortality [15] and deworming on school attendance [16]. The experimental evaluation should be conducted in a pragmatic setting with organizations already providing TB treatment and monitoring outcomes in order to mimic a real-world policy change, improve the generalizability of results and minimize the Hawthorne effect. To minimize any contamination of the control group, facilities (rather than individual patients) could be randomized to DOT vs. self-administration. Because DOT imposes greater costs on providers and patients, demonstrating the non-inferiority of self-administration relative to DOT is sufficient to motivate changes in TB program treatment protocol.

Each aspect of TB treatment should be designed to cure TB, prevent relapse, and minimize the emergence of drug resistance in the most effective and cost-effective way possible. Rigorously evaluating one or more of the following components in a real-world setting would build the evidence base to inform the optimal design of the TB treatment program. First, TB patients could *self-administer treatment* under supervision of a health care worker or use an alternative DOT observer such as a community leader [6]. Family members are unlikely to be the ideal observers. This could increase patient autonomy and self-efficacy while minimizing the need for frequent health facility visits. Second, an evaluation could *establish the optimal frequency* of health care worker visits to provide necessary patient support, screen for side effects and promptly identify barriers to adherence, while minimizing the burden on patients [17]. Third, patient and peer *support groups* could improve patient education and provide support for adherence and treatment completion [18]. Fourth, the *development of patient-centered training and educational materials* could increase patient treatment literacy for self-administering treatment. Finally, a *focus on patient-centered delivery of treatment* could improve outcomes for all TB patients. DOT proponents have argued that requiring DOT is necessary to promote early detection of treatment failure and engender effective patient-provider communication [19]. However, neither of these goals require DOT, and can be achieved with self-administration or alternative versions of TB treatment. Ideally, a series of rigorous RCTs, conducted in multiple countries and in a variety of operational settings, would evaluate these components and build a robust evidence base for TB policy and practice. These studies could also be accompanied by a rigorous costing analysis.

## Conclusion

Some proponents of direct observation have argued that altering TB treatment, such as allowing deviations from DOT, is a “slippery slope” that will ultimately lead to an erosion of support for TB control and a reduction in quality of care [20]. However, the design of TB programs must remain responsive to new developments in the epidemic and the evidence base. It is time to invest in the rigorous evaluation of DOT to determine whether the additional cost of this practice is justified by improved outcomes for TB patients worldwide. We call upon the implementing community to affirm its commitment to evidence-based policy making and upon the research community to produce high-quality evidence to support these critical decisions. Maintaining the status quo in the absence of rigorous supportive evidence may diminish the effectiveness of TB control in the long run.

## Abbreviations

DOT: Directly observed therapy
DS-TB: Drug susceptible TB
TB: Tuberculosis
